# Hypersensitivity Pneumonitis: Clinical Paradigm Evolution, Immune‐Driven Mechanisms, and Fibrotic Progression

**DOI:** 10.1155/jimr/1288887

**Published:** 2026-11-06

**Authors:** Chengsheng Yin, Yanrui He, Ping Wang, Zuojun Xu

**Affiliations:** ^1^ Department of Pulmonary and Critical Care Medicine Peking Union Medical College Hospital Chinese Academy of Medical Sciences and Peking Union Medical College Beijing China cacms.ac.cn; ^2^ Department of Pulmonary and Critical Care Medicine Shanghai Pulmonary Hospital Tongji University School of Medicine Shanghai China tongji.edu.cn

**Keywords:** antigen-driven fibrosis, fibrotic HP, gene–environment interaction, hypersensitivity pneumonitis, Th1/Th17-to-Th2/M2 immune polarization

## Abstract

Hypersensitivity pneumonitis (HP) is a complex immune‐mediated interstitial lung disease (ILD) triggered by inhaled antigens in genetically susceptible individuals. Recent diagnostic paradigms have shifted from exposure‐centric empiricism to fibrosis‐based stratification and, most recently, separation of etiology from morphology, with fibrotic HP (FHP) now recognized as a progressive fibrotic phenotype distinct from yet overlapping with idiopathic pulmonary fibrosis (IPF). Mechanistically, HP progression reflects a sequential cascade involving gene–environment interactions, dysregulated innate and adaptive immunity, and maladaptive repair. Early disease is characterized by Th1/Th17‐driven granulomatous inflammation, whereas chronic FHP exhibits Th2/M2 polarization, tertiary lymphoid structures, B‐cell dysregulation, cellular senescence, lung microbiome dysbiosis, and persistent fibroblast activation, ultimately leading to progressively less reversible remodeling. Despite shared terminal pathways with IPF (e.g., extracellular matrix (ECM) deposition, EndoMT, and SASP), FHP may be conceptualized as predominantly inflammation‐driven or “top‐down,” whereas IPF is often viewed as predominantly epithelial injury‐driven or “bottom‐up”; however, these heuristic models are not mutually exclusive and do not establish a discrete mechanistic transition or a uniform therapeutic window. Antigen avoidance remains the cornerstone of management; immunomodulatory treatment may be considered in selected inflammation‐predominant phenotypes, whereas antifibrotic treatment may be considered when FHP meets criteria for progressive pulmonary fibrosis. The role of combined therapy remains uncertain, and strategies targeting IL‐17, IL‐6, or JAK‐STAT remain investigational in HP. Early diagnosis is hindered by occult antigens and nonspecific presentations. Future advances will depend on multiomics integration, novel antigen‐detection tools, and biomarkers that may help characterize the proposed transition from predominantly antigen‐responsive inflammation toward fibrosis‐dominant remodeling. Resolving whether a clinically meaningful transition exists may support a more mechanism‐informed approach to HP. Unlike previous reviews focusing on diagnostic criteria, this review integrates our interpretation of the morphology–etiology distinction emphasized by the 2025 ERS/ATS classification update with single‐cell‐resolved immune evidence and uses the “immunofibrotic switch” as an integrative hypothesis rather than an established biological transition.

**Summary**

• Contemporary multidisciplinary assessment distinguishes morphological pattern description from etiological diagnosis and prioritizes the fibrotic extent and longitudinal behavior for prognostication.
• Pathogenesis involves gene–environment interactions and a proposed shift from inflammation‐predominant disease toward increasingly persistent fibrotic remodeling.
• Fibrotic hypersensitivity pneumonitis (FHP) and idiopathic pulmonary fibrosis (IPF) differ in the relative prominence of upstream immune and epithelial‐repair mechanisms, but these pathways overlap, and treatment responsiveness is stage‐ and phenotype‐dependent.

## 1. Introduction

Hypersensitivity pneumonitis (HP) comprises a spectrum of complex, immune‐mediated interstitial lung diseases (ILDs) triggered by the inhalation of sensitizing antigens [1–3]. According to the 2020 ATS/JRS/ALAT guidelines, the pathogenesis of HP arises from a dynamic interaction between environmental antigen exposure and host genetic susceptibility [3]. Ranking as the third most prevalent ILD, following idiopathic pulmonary fibrosis (IPF) and connective tissue disease‐associated ILD (CTD‐ILD) [4, 5], HP has an estimated annual incidence of 3–30 per 100,000 [6–8]. Clinical observations reveal significant epidemiological heterogeneity driven by geographic diversity, diagnostic discrepancies, and socioeconomic disparities.

Clinically, the presentation of HP lacks specificity. While traditionally categorized by duration (acute, subacute, and chronic), contemporary perspectives emphasize the presence of fibrosis as the critical determinant of prognosis. Patients with early or nonfibrotic disease typically present with cough, dyspnea, and flu‐like symptoms [4, 6]. In contrast, progression to fibrotic HP (FHP) manifests as worsening dyspnea, reduced exercise tolerance, and persistent pulmonary impairment, exhibiting a clinical trajectory highly analogous to that of IPF [9, 10]. Consequently, structural remodeling, rather than disease duration, is increasingly recognized as the core metric for characterizing disease severity and stratifying management [11].

Although HP has been recognized as a clinical entity for decades, the core pathogenic mechanisms governing the transition from antigen‐triggered immune inflammation to self‐sustaining fibrotic remodeling remain incompletely elucidated [1, 12]. Current research suggests a continuous, dynamic process: the host genetic background establishes a susceptibility baseline, complex antigen exposure triggers the immune response, and the interplay between innate and adaptive immune cascades drives tissue injury and aberrant repair [13, 14].

Given this background, this review first outlines the conceptual evolution of HP, ranging from an “exposure‐centric diagnosis” to “fibrosis‐based stratification” and the recently proposed pattern of bronchiolocentric interstitial pneumonia (BIP) [15, 16]. Subsequently, the key molecular networks underlying “gene‐environment‐immune” interactions are systematically summarized. Finally, particular attention is directed toward the core mechanisms driving the transition to fibrotic phenotypes, aiming to provide new perspectives for precision diagnosis and targeted therapy.

## 2. Evolution of Diagnostic Paradigms in HP: From Empiric Exposure to Etiology–Morphology Separation (Figure 1)

Over the past five decades, the conceptual framework of HP has undergone profound transformation, evolving from empiric exposure‐based diagnosis to a sophisticated model that integrates structural remodeling severity, prognostic stratification, and, most recently, separation of etiology from morphology [6, 17, 18].

**Figure 1:**
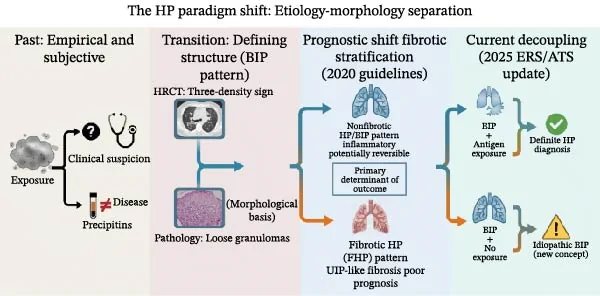
Morphology–etiology distinction in the multidisciplinary assessment of hypersensitivity pneumonitis. This diagram presents our interpretation of the morphology–etiology distinction emphasized by the 2025 ERS/ATS classification update; it is not an HP‐specific diagnostic rule. Bronchiolocentric interstitial pneumonia (BIP) is a descriptive pattern that is not specific for HP. Neither BIP morphology nor a reported exposure alone establishes definite HP; diagnosis requires integration of exposure assessment, HRCT, BAL, histopathology, diagnostic confidence, and alternative causes. Failure to identify an antigen does not by itself exclude HP, whereas idiopathic BIP should be considered only as a provisional diagnosis after comprehensive evaluation fails to identify an underlying cause. BIP, bronchiolocentric interstitial pneumonia; FHP, fibrotic hypersensitivity pneumonitis; HP, hypersensitivity pneumonitis; HRCT, high‐resolution computed tomography; UIP, usual interstitial pneumonia.

### 2.1. Early Era: Exposure‐Driven Empiric Diagnosis

HP was originally recognized as an occupation‐ or hobby‐related disorder, exemplified by classic entities such as farmer’s lung and bird fancier’s lung [17]. Diagnosis relied heavily on a compatible exposure history coupled with respiratory symptoms. The advent of precipitin testing (antigen‐specific IgG) provided the first serological evidence [10, 19]; however, it soon became evident that antibody positivity does not equate with disease, as many highly exposed yet asymptomatic individuals remain seropositive [20]. In the absence of objective imaging or histopathology, distinguishing causative from incidental exposure proved a persistent challenge.

### 2.2. Imaging Revolution and Recognition of the Bronchiolocentric Pattern

Diagnostic advances have shifted HP evaluation from exposure history dependence to integrated pattern recognition using histopathology and HRCT [21, 22]. Since the early 2000s, widespread HRCT adoption and minimally invasive bronchoscopic sampling have enabled precise identification of the hallmark “three‐density sign”—concurrent ground‐glass opacities (alveolar inflammation), air‐trapping (small airway obstruction), and spared parenchyma within the same secondary lobule—accurately reflecting HP’s mixed interstitial‐bronchiolocentric pathology [23, 24]. These HRCT features correlate closely with histology, where guidelines highlight poorly formed noncaseating granulomas as a core criterion. Acute HP shows neutrophil‐rich bronchiolitis with peribronchiolar lymphocytic infiltrates, while chronic HP exhibits septal thickening and fibrosis, occasionally with usual interstitial pneumonia (UIP)‐like foci and collagen deposition—features linked to reduced transplant‐free survival and steroid resistance [10, 11]. Chronic cases display reticular opacities and traction bronchiectasis along bronchovascular bundles, contrasting with UIP’s subpleural‐basal predominance and aiding IPF differentiation [11]. This pattern is now termed BIP, encompassing the classic histologic triad of cellular bronchiolitis, loose granulomas, and air trapping [16].

### 2.3. Prognostic Reorientation: Introduction of Fibrotic Stratification

Long‐term observational studies have shown that some patients historically classified as having chronic HP develop a fibrotic phenotype with a mortality risk approaching that of IPF [25, 26], challenging the traditional view of HP as a predominantly inflammatory and potentially reversible disorder [11]. Accordingly, the 2020 ATS/JRS/ALAT and 2021 CHEST guidelines adopted a phenotype‐based classification distinguishing non‐FHP from FHP, replacing the conventional acute/subacute/chronic framework [9, 10]. This shift established the presence and extent of fibrotic remodeling, rather than symptom duration alone, as major determinants of clinical outcome and positioned FHP within the broader spectrum of progressive fibrotic ILDs [5]. Nevertheless, fibrotic status alone does not fully capture the prognostic heterogeneity of FHP because the morphological pattern of fibrosis may provide additional prognostic information. In particular, UIP‐like morphological features may help identify patients at increased risk of functional decline and mortality [11, 24, 25, 27, 28], as discussed in detail in Section 5.4. Thus, contemporary prognostic assessment of HP should consider not only the presence and extent of fibrosis but also its specific radiological and histopathological pattern.

### 2.4. The 2025 ERS/ATS Classification Update: Distinguishing Morphological Patterns From Etiological Diagnoses

The 2025 ERS/ATS statement is an official consensus update to the international multidisciplinary classification of interstitial pneumonias rather than a dedicated HP diagnostic guideline. It recognizes BIP as a major morphological pattern and distinguishes descriptive radiological or histopathological patterns from final multidisciplinary diagnoses [16]. In this review, we refer to this distinction as an “etiology–morphology separation” framework; this term represents our interpretation of the classification update rather than formal terminology or a specific diagnostic recommendation from the statement.

BIP may occur in HP and other clinical contexts and therefore neither establishes nor excludes HP by itself. HP diagnosis should continue to integrate exposure assessment, HRCT, BAL, histopathology, diagnostic confidence, and exclusion of alternative causes through multidisciplinary discussion [9, 10, 20]. Failure to identify a causative antigen does not preclude HP, whereas “idiopathic BIP” may be considered provisionally when no underlying cause is identified after a comprehensive evaluation [16]. Thus, the 2025 statement provides a more precise morphological vocabulary but does not replace existing HP guidelines; the distinction between morphology and etiology should be viewed as a useful interpretative framework rather than a universally established HP‐specific diagnostic paradigm.

## 3. Pathogenesis: From Immune Dysregulation to Fibrotic Remodeling (Figure 2)

HP is an immune‐mediated lung disease triggered by inhaled antigens in susceptible individuals. Current evidence supports interactions among exposure, genetic background, innate and adaptive immunity, epithelial injury, and maladaptive repair. The evidence summarized in Sections 2 and 3 is heterogeneous: human cohorts, BAL, and histopathological studies mainly establish clinical and cellular associations; single‐cell studies identify disease‐associated states; and much of the causal or temporal evidence derives from animal models, in vitro systems, or extrapolation from other fibrotic ILDs. The proposed sequence from inflammation to fibrotic remodeling should therefore be regarded as an integrative hypothesis rather than a validated pathway in the human HP.

**Figure 2:**
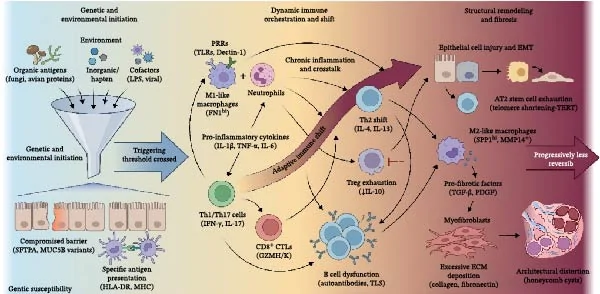
Integrated pathogenesis of hypersensitivity pneumonitis: from susceptibility and exposure to fibrotic remodeling. The schematic presents a hypothesis‐generating sequence across three conceptual phases. Left (initiation): cumulative environmental exposures may cross a triggering threshold in genetically susceptible individuals. Middle (immune orchestration): human and experimental studies identify inflammatory states involving FN1‐high macrophages and Th1/Th17‐associated responses, while Th2‐associated signaling, regulatory‐cell dysfunction, and B‐cell abnormalities have been proposed in fibrotic disease. Right (remodeling): epithelial injury, impaired regeneration, profibrotic macrophage states, and fibroblast–matrix interactions may contribute to ECM deposition and architectural distortion. This sequence integrates heterogeneous human and experimental evidence and should not be interpreted as a uniform, discrete, or irreversible transition in human HP. AT2, alveolar type 2 epithelial cell; ECM, extracellular matrix; EMT, epithelial‐mesenchymal transition; FN1, fibronectin 1; HP, hypersensitivity pneumonitis; LPS, lipopolysaccharide; MUC5B, mucin 5B; PDGF, platelet‐derived growth factor; SFTPA, surfactant protein A; SPP1, secreted phosphoprotein 1 (osteopontin); TGF‐β, transforming growth factor‐beta; TLR, Toll‐like receptor.

### 3.1. Genetic Susceptibility

Although HP usually presents sporadically, only a minority of similarly exposed individuals develop the disease, supporting a role for host susceptibility. Human association studies suggest a polygenic architecture involving antigen presentation, epithelial barrier function, and repair; however, the contribution of individual variants varies across populations and does not establish a deterministic threshold for disease.

Human genetic association studies have linked selected surfactant‐protein variants, including SFTPA1 and SFTPB polymorphisms, with HP susceptibility in specific populations [14]. Proposed effects on surfactant assembly, antigen clearance, and macrophage regulation are biologically plausible, but the causal mechanisms have not been directly demonstrated in human HP.

MHC/HLA variation can alter peptide presentation and immune‐response thresholds [29, 30]. Associations with HP have been reported for HLA‐DR3, HLA‐DR7, and selected DRB1 alleles in population‐ and exposure‐specific settings [31–34], while antigen‐processing genes such as PSMB8 and TAP1 have also been implicated [35, 36]. These findings support genetic susceptibility but should not be interpreted as universal causal markers.

The genetic architecture of FHP partially overlaps with that of other fibrotic ILDs. In human observational cohorts, the MUC5B promoter variant and shorter telomere length have been associated with fibrotic phenotypes and adverse outcomes [37, 38]. Proposed links to mucociliary dysfunction, epithelial‐cell senescence, and impaired repair are supported largely by mechanistic work in other fibrotic disorders and require direct validation in HP.

Available evidence is consistent with a gene–environment interaction rather than either factor acting alone. Exposure‐specific HLA associations and variable disease penetrance illustrate this principle, although the antigen‐specific molecular interactions remain incompletely characterized [39, 40].

### 3.2. Antigen Exposure and Environmental Triggers: Initiation of the Immune Cascade

The physicochemical properties and exposure pattern of environmental antigens serve as the trigger for the HP immune cascade [7]. Different antigen types are recognized by specific pattern‐recognition receptors (PRRs), thereby determining the nature of the subsequent inflammatory response and the resulting pathological phenotype [41, 42].

Different exposure classes are associated with variable clinical and pathological phenotypes, but mechanistic attribution remains incomplete [43, 44]. Evidence that fungal β‐glucan signals through Dectin‐1 and that microbial CpG‐DNA or lipopeptides activate TLR pathways derives mainly from experimental systems [45–47]. Likewise, proposed inflammasome activation by chemicals or metal‐associated exposures is supported primarily by preclinical and translational observations rather than direct causal evidence in human HP [48, 49].

Experimental models indicate that coexposures can modify immune polarization. For example, fungal antigens combined with lipopolysaccharide can favor IL‐17A‐dominant inflammation and giant‐cell formation in animal models [46]. Whether comparable adjuvant effects determine the rate of progression in human HP remains uncertain.

Smoking has long been associated with a lower incidence of clinically recognized HP yet with poorer outcomes once FHP is established—a pattern often termed the “smoking paradox.” One proposed explanation is that nicotine activates the α7 nicotinic acetylcholine receptor (α7‐nAChR) pathway, suppressing proinflammatory cytokine release by alveolar macrophages and attenuating Th1/Th17 responses, potentially favoring a relative Th2 bias [48, 50–52]. This effect may dampen early antigen‐driven inflammation. Prolonged α7‐nAChR signaling has also been proposed to reduce Treg‐derived IL‐10 and contribute to later immune dysregulation, a possibility that may relate to the proposed concept of Treg exhaustion [49]. These mechanistic links derive largely from animal and in vitro studies and have not been established as a temporal sequence in human FHP. The observed lower incidence of clinically recognized HP among smokers, therefore, should not be interpreted as proof that smoking is protective, nor does it establish a uniform late Treg defect. Attenuated early symptoms could plausibly delay clinical recognition in some patients, allowing fibrosis to be present at the diagnosis, but this explanation also requires direct clinical validation [50].

### 3.3. Immune Pathogenesis: From Inflammatory Storm to Fibrotic Remodeling

The pathological progression of HP results from the dynamic interaction between innate and adaptive immunity, displaying clear spatiotemporal characteristics: from early‐stage Th1/Th17‐driven granulomatous inflammation to later‐stage Th2/M2‐dominant fibrotic remodeling.

#### 3.3.1. Innate Immunity: Initiation of Inflammation

Innate immunity serves not only as the initiator of inflammation but also as a central hub that determines the direction of adaptive immunity through cytokine secretion and antigen presentation [53, 54].

##### 3.3.1.1. Signal Cascade Amplification

Experimental HP models show that PRRs, including TLR2/6 and Dectin‐1, can activate MyD88/NF‐κB and MAPK signaling and increase IL‐1β, TNF‐α, and IL‐6 production [47, 55, 56]. Direct confirmation of the relative contribution of these pathways across human HP phenotypes remains limited.

##### 3.3.1.2. Inflammasome Activation and Upstream Cytokines

Preclinical and broader lung injury studies suggest that assembly and activation of the NLRP3 inflammasome promote the proteolytic maturation and release of IL‐1β and IL‐18, which may help sustain early neutrophilic inflammation and create a cytokine milieu conducive to subsequent Th17 differentiation [57–59]. Complement activation may further amplify these responses: C3a and C5a can recruit inflammatory cells, while locally generated C3b may enhance dendritic cell antigen presentation and facilitate T cell activation [57, 58]. Together, these pathways offer a plausible link between innate inflammatory responses and adaptive immunity in HP. However, their causal importance, timing, and contribution to progression in human HP remain to be established.

##### 3.3.1.3. Phenotypic Shift in Mononuclear‐Phagocyte System

Of particular interest is the diversity of macrophage states observed in FHP. Human single‐cell studies have identified FN1‐high inflammatory macrophages and SPP1‐high profibrotic macrophages, with PLA2G7‐high populations also described in the fibrotic setting [60–62]. These findings are consistent with a model in which macrophages contribute to both antigen‐related inflammatory responses and fibrotic remodeling. However, the available human data are largely cross‐sectional: the coexistence of these populations does not establish that FN1‐high cells transition over time into PLA2G7‐high or SPP1‐high cells [61, 62]. Profibrotic macrophage transcriptional programs are compatible with the production of mediators such as CCL18, TGF‐β, and PDGF, which may support fibroblast activation and extracellular matrix (ECM) deposition; their direct contribution to these processes, and to epithelial–mesenchymal transition in human FHP remains uncertain [63, 64]. Separately, preclinical evidence from a *Saccharopolyspora rectivirgula* antigen (SR‐Ag) mouse model identified an MMP14‐high macrophage population with M2‐like features. In that model, TLR2/NF‐κB‐regulated MMP14 expression and exosome‐associated signaling were implicated in the fibroblast‐to‐myofibroblast transition [65]. Together, these observations support macrophages as a possible link between inflammation and fibrosis, while the proposed temporal shift and MMP14‐dependent mechanism require validation in the human HP.

##### 3.3.1.4. Aberrant Innate Immune‐Related Gene Expression in Fibroblasts and Profibrotic Effects

In vitro studies using fibroblasts isolated from patients with HP have identified altered expression of innate immune response genes, including increased IFI27 and PDGFRA, reduced IL17RC and TGFBR3, and a shift in the relative expression of soluble versus full‐length AGER isoforms [66]. IFI27 is associated with type I interferon signaling, suggesting that inflammatory cues may influence fibroblast behavior. In these experimental systems, manipulation of IFI27 affected myofibroblast differentiation, including *α*‐smooth muscle actin expression, as well as cell proliferation over time, supporting a potential profibrotic role. Increased PDGFRA expression may also alter responsiveness to growth factor signals, although the functional consequences of the other expression changes remain less clear. Together, these findings suggest that HP fibroblasts may respond actively to innate immune signals rather than solely deposit the ECM. Because the functional evidence comes from cultured cells, it does not establish how strongly these pathways operate in vivo, when they emerge during HP progression, or whether they reinforce macrophage‐driven fibrosis in patients [66].

#### 3.3.2. Adaptive Immunity: The Dominant Force in Disease Progression

##### 3.3.2.1. Dynamic Balance and Functional Remodeling of T‐Cell Subsets

HP is regarded as a combined pathological process of type III (immune complex‐mediated) and type IV (T‐cell‐mediated) hypersensitivity. However, deeper mechanistic studies show that shifts in T‐cell subsets may contribute to progression from granulomatous inflammation toward fibrosis‐dominant remodeling. As central effector cells of adaptive immunity, different T‐cell subsets (Th1, Th17, Th2, cytotoxic T cells [CTLs], and Tregs) exhibit a complex dynamic balance and functional remodeling over time and space.

##### 3.3.2.2. Th1 Cells: Initiators of Granulomatous Inflammation

Human BAL and lung tissue studies support prominent Th1‐associated signals in inflammatory HP, including increased T‐cell markers such as CD3D, CD8, and granzyme A, as well as IFN‐γ‐associated chemokines such as CXCL9, CXCL10, and CCL5 [53, 67, 68]. These observations are consistent with recruitment of CXCR3‐expressing T cells and potentially NK cells, to affected lung regions. Experimental models, rather than direct longitudinal studies in patients, suggest that repeated antigen exposure activates dendritic cells and Th1‐associated cytokine responses, including IFN‐γ and IL‐12, which can promote granulomatous inflammation and inflammatory cell recruitment [69, 70]. Population‐specific HLA associations may influence antigen recognition and susceptibility, but the proposed sequence linking a particular HLA variant to increased T‐cell receptor affinity and CXCL9/CXCL10‐mediated recruitment has not been demonstrated directly in patients with HP [14].

##### 3.3.2.3. Th17 Cells: A Bridge Between Chronic Inflammation and Fibrosis

Murine HP models indicate that IL‐23 and IL‐1β signaling can favor Th17 differentiation and that IL‐17A can amplify neutrophilic inflammation and fibrotic responses after repeated antigen exposure [56, 71–74]. Experimental findings have also raised the possibility of ITK‐independent Th17 polarization under strong thermophilic actinomycete‐associated inflammatory stimulation; whether this occurs in human HP or alters its clinical course remains unknown. At the effector level, proposed pathways include NF‐κB‐associated CXCL5 induction and neutrophil recruitment through the IL‐17A–CXCL5 axis. IL‐17‐related effects on epithelial integrity, TGF‐β‐associated signaling, and fibroblast activation, potentially involving IL‐22, are also supported by translational studies in other fibrotic ILDs [75]. Together, these findings provide a rationale for a role of Th17‐associated inflammation in tissue remodeling, but they do not establish a uniform Th17‐mediated progression to fibrosis or a defined sequence of epithelial–mesenchymal transition in human FHP.

##### 3.3.2.4. Th2 Cells: Drivers of the Fibrotic Phenotype Conversion

Th2‐skewed responses and M2‐like macrophage programs have been observed in experimental chronic HP and in selected human fibrotic samples [49, 76]. In experimental systems, IL‐4/IL‐13–STAT6 signaling can promote profibrotic macrophage and fibroblast responses. Proposed downstream effects include epithelial remodeling and the production of mediators such as PDGF, FGF2, and tissue inhibitors of metalloproteinases, which may favor fibroblast proliferation and ECM accumulation. However, the suggested sequence in which prolonged antigen exposure reduces dendritic‐cell IL‐12 production, weakens Treg‐mediated suppression, and shifts the response from Th1/Th17 toward Th2 remains a proposed model. Neither this temporal transition nor its proposed relationship to irreversible fibrosis has been validated in human HP. Telomere dysfunction, including TERT‐related abnormalities, and impaired epithelial repair may further modify susceptibility to fibrotic progression, but current evidence does not identify a discrete point at which inflammation becomes irreversible fibrosis [38, 77].

##### 3.3.2.5. CTLs and Regulatory Cell Imbalance

Human single‐cell transcriptomic studies have identified expanded effector‐memory CD8^+^ T‐cell populations expressing GZMH and GZMK in FHP [62, 78]. Their transcriptional profiles suggest possible cytotoxic and macrophage‐modulating functions but do not demonstrate that these cells sustain granulomatous inflammation or drive fibrotic progression. Some transcriptional features overlap with those reported in systemic lupus erythematosus; this is a descriptive cross‐disease comparison, not evidence of a shared pathogenic mechanism [79]. Treg dysfunction has been proposed as a contributor to impaired immune regulation, although direct evidence of Treg exhaustion in human FHP remains limited and is partly extrapolated from IPF and basic immunology [80, 81]. BAL studies have also described variation in NK and NKT cells during lymphocytic alveolitis, a finding that is not specific to FHP [82]. γδ T cells and tissue‐resident memory T cells may participate in local immune responses, but their specific profibrotic functions in human HP remain incompletely defined [76].

#### 3.3.3. Functional Evolution of B Cells: From Antigen Presentation to Ectopic Lymphoid Tissue

Experimental models and human tissue studies suggest that B‐cell functions in HP are heterogeneous and context‐dependent. B‐cell depletion or gene‐knockout experiments in selected models have not uniformly suppressed inflammation [45, 83], whereas other exposure systems and human histopathology show B‐cell accumulation and tertiary lymphoid structures [48, 84, 85]. The proposed temporal evolution from lymphoid cuffs to organized lymphoid structures and plasma‐cell‐rich fibrotic lesions remains incompletely validated in longitudinal human disease.

B cells may contribute through antigen presentation, cytokine signaling, and humoral responses in addition to antibody production [86–89]. Experimental studies show that IL‐10‐producing regulatory B cells can modulate T‐helper responses [90], but direct evidence for progressive B10 exhaustion, BAFF/APRIL‐driven fibroblast activation, or a causal IL‐6/STAT3 B‐cell pathway in human FHP is insufficient. These mechanisms should therefore be viewed as testable hypotheses rather than established drivers of persistent fibrosis.

In essence, the pathogenesis of HP may be conceptualized as a genetically modulated continuum involving interactions among inhaled antigens, innate and adaptive immune responses, epithelial injury, and tissue‐repair mechanisms. In some patients, persistent disruption of immune and epithelial homeostasis may promote a gradual shift from predominantly antigen‐responsive inflammation toward increasingly self‐sustaining fibrotic remodeling. Within this proposed framework, disease progression may become partially uncoupled from the initial antigenic stimulus through putative mechanisms such as fibroblast epigenetic reprogramming and regulatory T‐ and B‐cell dysfunction. We use the term “immunofibrotic switch” as a hypothesis‐generating conceptual framework to integrate these observations, rather than to denote a discrete or experimentally demonstrated biological event. An important priority for future research is therefore to identify and validate biomarkers that can determine whether such a clinically meaningful transition exists, define its temporal and biological boundaries, and establish whether it represents a therapeutically modifiable stage of the disease.

## 4. Drivers of Chronicity and Fibrotic Progression in HP (Figure 3)

In some patients with chronic HP, fibrotic remodeling progresses despite the reduction or removal of the suspected exposure. Current evidence supports potential contributions from persistent immune signaling, epithelial senescence, altered tissue repair, microbiome‐associated signals, and sustained fibroblast activity; however, a fully antigen‐independent, self‐perpetuating loop has not been demonstrated in human HP [64]. These factors are therefore considered candidate drivers of chronicity rather than a proven irreversible sequence.

**Figure 3:**
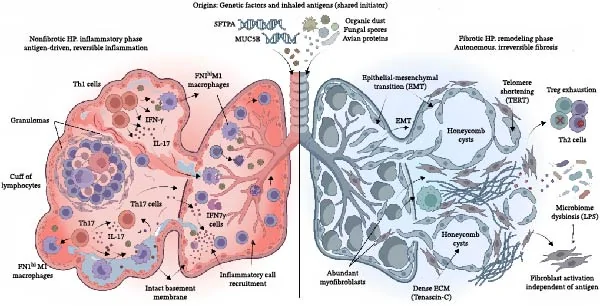
Inflammatory and fibrotic phenotypes of hypersensitivity pneumonitis. While both phenotypes share common triggers (inhaled antigens and genetic susceptibility), they exhibit distinct biological behaviors. Nonfibrotic HP (left) is defined by an active, inflammatory cell recruitment phase involving granuloma formation and specific macrophage subsets (FN1^hi^ M1), where the lung architecture remains largely preserved. In contrast, fibrotic HP (right) is characterized by structural remodeling that may become increasingly self‐sustaining in some patients. This phase is driven by epithelial injury (EMT, telomere dysfunction), immune dysregulation (Treg exhaustion), and microbiome changes, leading to extensive myofibroblast activation and honeycombing that may continue despite removal or avoidance of the suspected inciting antigen. ECM, extracellular matrix; EMT, epithelial‐mesenchymal transition; HP, hypersensitivity pneumonitis; IFN‐γ, interferon‐gamma; LPS, lipopolysaccharide; MUC5B, mucin 5B; SFTPA, surfactant protein A; Th1/Th17/Th2, T helper cell subsets; Treg, regulatory T cell.

### 4.1. Host Factors and Comorbid Features: Reshaping Prognosis

Observational HP cohorts have associated older age, shorter telomeres, autoimmune features, hypothyroidism, smoking history, and fibrotic morphological patterns with disease severity or adverse outcomes, although the strength of these associations varies by population and endpoint [8, 11, 27, 40, 91–93]. Age‐related immune changes and telomere dysfunction may jointly reduce epithelial repair capacity and alter T‐cell regulation, potentially contributing to more extensive fibrosis. However, the proposed sequence from immunosenescence to T‐cell exhaustion, Treg dysfunction, honeycombing, and shortened survival has not been demonstrated directly in patients with HP. Autoimmune features, including elevated antinuclear antibodies or rheumatoid factor, may coexist with HP; epitope spreading and TGF‐β‐related signaling are possible explanations rather than established causes of progression. Hypothyroidism has also been associated with adverse outcomes in some cohorts, but its independent prognostic contribution requires interpretation in the context of each study’s adjustments and endpoint. Likewise, although smoking has been associated with a lower incidence of clinically recognized HP, smoking history or continued exposure may be associated with worse outcomes once fibrotic disease is established. These clinical associations should not be taken as proof of the proposed immune mechanisms.

### 4.2. Persistent Fibroblast Activation and ECM Imbalance

In chronic FHP, persistent fibroblast activation may be supported by profibrotic signals from immune cells, altered interactions with the ECM, and recruitment of circulating fibrocytes [76, 94]. Experimental studies suggest that IL‐13 and TGF‐β signaling, as well as matrix‐associated proteins such as tenascin‐C and periostin, can reinforce fibroblast survival and matrix production through biochemical and mechanical cues. Meanwhile, human BAL, tissue, and genetic studies have reported changes in matrix‐associated proteins, metalloproteinases, including MMP‐8 and MMP‐9, and their inhibitors, including TIMP3 [95, 96]. These findings are consistent with dysregulated matrix turnover and a fibrotic microenvironment that may help sustain remodeling. Nevertheless, the contributions of individual pathways to FHP progression remain incompletely defined, and neither fully autonomous fibroblast activity nor uniformly irreversible ECM deposition has been demonstrated in human HP.

### 4.3. Microbiome Dysbiosis as a Potential Modifier of Fibrotic Progression

Human observational studies have identified differences in lower‐airway bacterial community composition between chronic HP and IPF [97]. Findings involving taxa such as Proteobacteria or Pseudomonas raise the possibility that microbial communities modify local inflammation, but these associations are sensitive to sampling, background exposures, and disease characteristics. Experimental studies in broader lung injury and fibrosis settings suggest that bacterial lipopolysaccharide can activate TLR4/NF‐κB signaling, increase inflammatory mediators such as IL‐6 and TNF‐α, and interact with TGF‐β‐related responses and MMP/TIMP‐regulated matrix turnover. Whether these pathways operate in the same way in human FHP remains unknown. In particular, current data do not establish that microbiome changes are antigen‐independent drivers of fibrosis, interact causally with HLA variation, or explain progression after antigen avoidance.

Chronic progression in HP may therefore reflect interactions among host susceptibility, impaired epithelial repair, persistent immune activity, fibroblasts, and the ECM. Microbiome variation is a plausible additional modifier, but its temporal and causal role requires prospective study in well‐phenotyped patients. Microbiome‐directed treatment should remain a research question until a clinically meaningful effect has been demonstrated [97].

## 5. Mechanistic Differences and Similarities Between FHP and IPF: A Comparative Pathogenetic Analysis (Figure 4)

FHP and IPF may share clinical, radiological, and prognostic features, particularly at the advanced stages. Bulk and single‐cell transcriptomic studies have identified overlapping programs related to ECM deposition, vascular remodeling, and endothelial‐to‐mesenchymal transition [12, 98, 99]. Extracellular‐vesicle signaling has been described in IPF, but its role as a shared driver of FHP remains unestablished [100]. These observations suggest that the two diseases may converge on some downstream remodeling pathways while differing in their identifiable triggers and the relative prominence of immune and epithelial injury.

**Figure 4:**
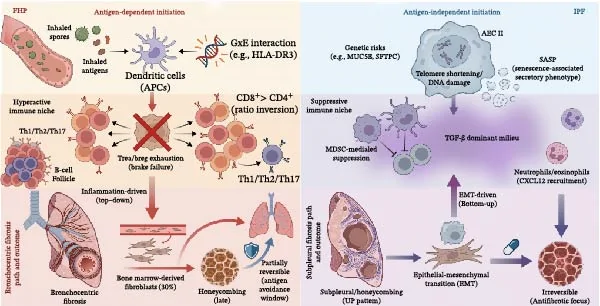
FHP vs. IPF: distinct upstream drivers converging on pulmonary fibrosis. This schematic presents the proposed “top‐down” and “bottom‐up” models as heuristic representations of the relative predominance of immune‐mediated injury in FHP and epithelial injury‐driven aberrant repair in IPF, respectively. In FHP, antigen‐associated immune activation may have a more prominent upstream role, whereas repetitive epithelial injury, cellular senescence, and impaired regeneration are generally more prominent in IPF. These pathways are not mutually exclusive, because immune activation, epithelial dysfunction, and mesenchymal remodeling may coexist in both diseases and increasingly overlap at advanced fibrotic stages. Although the two diseases may differ in their predominant initiating factors and treatment responsiveness, both can converge on shared profibrotic pathways and progressive structural remodeling. Accordingly, differences in reversibility should be regarded as stage‐ and phenotype‐dependent rather than absolute. AEC II, type II alveolar epithelial cell; APC, antigen‐presenting cell; EMT, epithelial‐mesenchymal transition; FHP, fibrotic hypersensitivity pneumonitis; GxE, gene–environment interaction; IPF, idiopathic pulmonary fibrosis; MDSC, myeloid‐derived suppressor cell; SASP, senescence‐associated secretory phenotype; TGF‐β, transforming growth factor‐beta; UIP, usual interstitial pneumonia.

### 5.1. Initiating Triggers and Disease Ontology

FHP is associated with immune responses to inhaled antigens in susceptible individuals. Human studies support associations involving environmental exposures, selected HLA alleles, and the MUC5B promoter polymorphism, although the magnitude and causal interpretation of individual gene–exposure interactions remain uncertain [37]. An exposure history is therefore an essential part of assessment but must be interpreted alongside radiological, BAL, and, when available, pathological findings.

IPF, by definition, is an idiopathic interstitial pneumonia without an identified cause. Its proposed pathobiology emphasizes recurrent alveolar epithelial injury, impaired regeneration, telomere dysfunction, cellular senescence, surfactant‐protein gene variants, and epigenetic changes [101, 102]. Innate immune responses may also modify epithelial injury and repair, but their timing and causal contribution remain incompletely defined. Antigen‐associated immune injury may be relatively more prominent upstream in FHP, whereas epithelial dysfunction is central to the prevailing models of IPF. This distinction may help explain some differences in clinical presentation and treatment approach, but it should not obscure the substantial mechanistic overlap between the diseases, especially after extensive fibrosis develops [103].

### 5.2. Pulmonary Immune Microenvironment

FHP and IPF differ in the relative composition of their pulmonary immune environments, although neither has a single, uniform immune profile. BAL lymphocytosis and T‐cell‐associated inflammation more often support HP, particularly in nonfibrotic or inflammation‐predominant disease. By contrast, IPF more commonly shows neutrophil‐ and monocyte‐associated signals [104, 105]. CD8^+^ T‐cell enrichment, altered regulatory‐cell activity, and Th1‐, Th17‐, Th2‐, or M2‐associated signatures have been reported in HP samples or experimental systems. However, these findings do not establish a consistent temporal transition from one immune state to another in individual patients with FHP.

These differences can inform diagnostic and therapeutic assessment, but immune‐cell profiles alone do not demonstrate that immunosuppression is effective in FHP. Evidence supporting corticosteroids and other immunosuppressants in HP remains predominantly observational, whereas chronic immunosuppression is generally avoided in IPF [106]. Treatment decisions in FHP should therefore consider the extent of active inflammation, established fibrosis, and observed disease behavior rather than rely on a proposed immune sequence.

### 5.3. Nature and Reversibility of Fibrosis

FHP may retain a substantial inflammatory component, particularly earlier in its course, and some patients may benefit from timely identification and avoidance of the inciting antigen. A proposed “top‐down” model describes how persistent antigen‐associated immune activity could contribute to fibroblast recruitment and activation, potentially with input from circulating fibrocytes [4]. FHP fibrosis is often bronchiolocentric on histopathology, although its distribution and accompanying features vary [16].

A proposed “bottom‐up” model of IPF instead emphasizes repeated alveolar epithelial injury, defective repair, and aberrant mesenchymal activation. IPF characteristically exhibits a UIP pattern with subpleural and basal predominance, fibroblastic foci, and honeycombing [107]. These models are useful for highlighting different predominant features, but they are heuristic rather than mutually exclusive mechanisms: epithelial injury, immune activity, and mesenchymal remodeling can occur in both diseases. Advanced fibrosis in either condition may become less responsive to the removal of an initiating stimulus or suppression of inflammation. Nevertheless, complete biological autonomy and uniform irreversibility have not been demonstrated in human FHP.

### 5.4. Prognostic Significance of the UIP‐Like Pattern in FHP

Radiological and histopathological studies have associated greater fibrotic extent, honeycombing, and UIP‐like features in FHP with adverse outcomes, including poorer transplant‐free survival and faster functional decline [11, 24, 25, 27]. Fibrotic burden is therefore important, but the morphological pattern may provide additional prognostic information.

In a multicenter retrospective cohort of 195 patients with FHP who underwent BAL and transbronchial lung cryobiopsy, Petrarulo et al. [28] identified a UIP‐like histopathological pattern in 59 patients (30%). Compared with patients without this pattern, those with UIP‐like morphology had lower BAL lymphocyte percentages (14.4% versus 37.4%), greater longitudinal declines in FVC and DLCO, and a higher 10‐year mortality risk (HR 2.8, 95% CI 1.3–5.8) [28]. These findings suggest that the UIP‐like morphology may identify a subgroup with a more progressive course. The lower BAL lymphocyte percentage is consistent with less prominent alveolar lymphocytosis but does not by itself prove a lower overall inflammatory burden or establish a specific mechanism.

A UIP‐like pattern in FHP is not equivalent to a diagnosis of IPF. Etiological attribution still requires integration of exposure history, HRCT, BAL, pathology, and multidisciplinary discussion. Moreover, the Petrarulo study was retrospective and restricted to patients selected for cryobiopsy. Its findings support closer prognostic attention to UIP‐like morphology but require prospective validation before this feature is used independently to determine treatment [28].

### 5.5. Clinical and Therapeutic Implications

The distinction among non‐FHP, FHP, and IPF informs both the diagnostic assessment and follow‐up. Fibrotic progression should be considered when worsening symptoms are accompanied by serial declines in FVC or DLCO, increasing oxygen requirements, or progression of reticulation, traction bronchiectasis, architectural distortion, or honeycombing on HRCT, particularly despite appropriate antigen avoidance. UIP‐like features in FHP warrant careful physiological and radiological monitoring because of their association with adverse outcomes, but they should not prompt automatic reclassification as IPF [11, 24, 25, 27, 28].

Diagnostic confidence should be established through multidisciplinary integration of exposure assessment, HRCT findings in both the lung parenchyma and small airways, BAL cellular profiles, and pathology when clinically indicated [9, 10, 104, 108]. BAL lymphocytosis can support HP, especially in nonfibrotic or inflammation‐predominant presentation. Conversely, a lower lymphocyte percentage may occur in advanced or UIP‐like FHP and does not exclude HP.

Management should be guided by the predominant clinical phenotype and longitudinal behavior. Identifying and avoiding the suspected inciting antigen remains fundamental, although evidence for its effect on long‐term outcomes comes mainly from observational cohorts [109]. Corticosteroids may be considered in patients with clinically important inflammatory disease, while mycophenolate mofetil or azathioprine may be used as steroid‐sparing agents in selected cases; adequately powered randomized HP trials have not established the efficacy of these approaches [109, 110].

Patients with objective progression despite appropriate management should be assessed for progressive pulmonary fibrosis. Nintedanib has the strongest randomized antifibrotic evidence in this setting, including patients with HP, although the HP subgroup was not independently powered to establish a treatment effect [111–113]. Evidence for pirfenidone in FHP remains less certain because a small randomized trial was underpowered for its primary endpoint [114]. Rituximab is supported only by small observational studies in refractory disease [115]. The optimal timing and efficacy of combined immunomodulatory and antifibrotic therapy remain uncertain. Strategies targeting IL‐17, IL‐6/IL‐6R, or JAK–STAT are investigational and should be distinguished from established treatment.

In IPF, antifibrotic treatment is an established pharmacological approach, whereas chronic immunosuppression is generally avoided. Supportive care, pulmonary rehabilitation, oxygen therapy, management of comorbidities, and timely consideration of lung transplantation should be incorporated according to the disease severity. Table 1 summarizes the principal phenotypic distinctions among non‐FHP, FHP, and IPF; Table 2 presents the evidence supporting established and investigational treatments.

**Table 1:** Clinical, radiological, pathological, immunological, prognostic, and therapeutic distinctions among nonfibrotic hypersensitivity pneumonitis, fibrotic hypersensitivity pneumonitis, and idiopathic pulmonary fibrosis.

Characteristic	Nonfibrotic HP	Fibrotic HP	IPF
Etiological context	Usually associated with an identifiable or suspected inhaled antigen, although the antigen may remain occult	Antigen may be identified, suspected, or remain unknown; fibrosis may progress despite exposure avoidance	Idiopathic; no defined inhaled‐antigen cause
Predominant pathological process	Inflammation‐predominant and bronchiolocentric	Mixed inflammatory and fibrotic remodeling; relative proportions vary among patients	Fibrosis‐predominant epithelial injury and aberrant repair
HRCT findings	Centrilobular ground‐glass nodules, mosaic attenuation, expiratory air trapping, ground‐glass opacity, and the three‐density pattern; no established fibrosis	Reticulation, traction bronchiectasis, architectural distortion, and possible honeycombing, frequently accompanied by mosaic attenuation, air trapping, or the three‐density pattern; UIP‐like appearances may occur	Basal and subpleural reticulation, traction bronchiectasis, and honeycombing consistent with a UIP or probable UIP pattern; extensive air trapping and the three‐density pattern are less typical
Histopathology	Cellular bronchiolitis, bronchiolocentric lymphocytic inflammation, and poorly formed nonnecrotizing granulomas	Bronchiolocentric fibrosis, airway‐centered metaplasia, bridging fibrosis, architectural distortion, and variable residual inflammation; fibroblastic foci and UIP‐like fibrosis may occur	Patchy subpleural and paraseptal fibrosis with temporal heterogeneity, fibroblastic foci, and honeycombing; granulomas and prominent bronchiolocentric inflammation are generally absent
BAL profile	Lymphocytosis is frequently prominent and supports the diagnosis	Lymphocytosis is variable and often lower than in nonfibrotic HP; particularly low levels may occur in UIP‐like or advanced fibrotic phenotypes	BAL lymphocytosis is generally absent or limited; BAL is used mainly to exclude alternative diagnoses
Immunological features	Antigen‐associated innate and adaptive immune activation, commonly involving Th1/Th17‐associated and granulomatous responses	Heterogeneous combination of persistent immune activation, regulatory‐cell dysfunction, profibrotic macrophage states, epithelial injury, and fibroblast activation; several proposed mechanisms remain incompletely validated in humans	Predominantly associated with epithelial dysfunction, cellular senescence, impaired regeneration, and mesenchymal activation, with immune pathways acting as potential disease modifiers
Reversibility	Frequently improves after successful antigen avoidance; corticosteroids may accelerate symptomatic or functional recovery in selected patients	Variable and generally more limited; inflammatory components may remain treatment‐responsive, whereas advanced structural fibrosis is less reversible	Established fibrosis is generally irreversible, although antifibrotic treatment may slow progression
Prognosis	Often favorable after complete antigen avoidance, although recurrence or subsequent fibrosis may occur	Heterogeneous; extent of fibrosis, honeycombing, physiological decline, and UIP‐like morphology are associated with worse outcomes	Usually progressive, with substantial morbidity and mortality
Principal management	Antigen identification and avoidance; observation or corticosteroids in symptomatic or clinically significant inflammatory disease	Antigen avoidance; phenotype‐directed consideration of immunomodulation when inflammatory activity is present; monitoring for progression and consideration of antifibrotic therapy in progressive pulmonary fibrosis	Antifibrotic therapy with nintedanib or pirfenidone, supportive care, management of comorbidities, and timely lung‐transplant assessment
Main evidence limitations	Limited randomized therapeutic evidence	Treatment evidence is mainly observational, except for antifibrotic evidence extrapolated from progressive pulmonary fibrosis trials; optimal combination treatment remains uncertain	Stronger randomized‐trial evidence for antifibrotic treatment, but substantial individual heterogeneity remains

*Note:* The distinctions shown are intended to summarize predominant features; substantial overlap exists, and final diagnosis should be established through multidisciplinary discussion. DLCO, diffusing capacity of the lung for carbon monoxide.

Abbreviations: BAL, bronchoalveolar lavage; FHP, fibrotic hypersensitivity pneumonitis; FVC, forced vital capacity; HP, hypersensitivity pneumonitis; HRCT, high‐resolution computed tomography; IPF, idiopathic pulmonary fibrosis; PPF, progressive pulmonary fibrosis; UIP, usual interstitial pneumonia.

**Table 2:** Evidence hierarchy for established and investigational therapies in HP.

Therapeutic strategy or target	Biological rationale	Preclinical or translational evidence	Clinical evidence in HP	Evidence level	Current clinical status
Antigen identification and avoidance	Removal of the suspected initiating exposure may reduce recurrent immune stimulation	Strong biological rationale; exposure‐response relationships supported by clinical and environmental studies	Observational cohorts associate complete avoidance with reduced recurrence and fibrosis in nonfibrotic HP and potentially improved outcomes in FHP	Level III	Foundational management strategy across HP phenotypes, although benefit may be less predictable in advanced FHP
Systemic corticosteroids	Broad suppression of inflammatory and immune responses	Supported by experimental immunology and established anti‐inflammatory effects	Retrospective cohorts suggest greater functional benefit in nonfibrotic or inflammation‐predominant HP; effects on survival and advanced FHP remain inconsistent	Level III	Commonly used in symptomatic inflammatory disease; not proven to prevent progression of established fibrosis
Mycophenolate mofetil or azathioprine	Steroid‐sparing suppression of lymphocyte proliferation and immune activation	General immunological rationale; limited HP‐specific mechanistic evidence	Retrospective cohorts report stabilization or improvement of DLCO in some patients, without consistent improvement in FVC or survival	Level III	May be considered in selected patients requiring longer‐term immunomodulation; not an established antifibrotic treatment
Rituximab/CD20^+^ B‐cell depletion	Reduction of B‐cell‐mediated antigen presentation, cytokine production, and humoral immune activity	Experimental studies support B‐cell involvement, although B‐cell functions in HP are heterogeneous	Small retrospective and prospective observational cohorts suggest possible stabilization in some treatment‐refractory patients	Level III, very low certainty	Rescue or investigational option in selected refractory cases; not standard therapy
Nintedanib	Inhibition of profibrotic receptor tyrosine kinases and fibroblast activity	Broad antifibrotic effects demonstrated in experimental fibrosis models	Randomized evidence from progressive fibrosing ILD trials, including an FHP subgroup, supports reduced FVC decline; HP‐specific subgroup analyses were not independently powered	Level II	May be considered in FHP meeting criteria for progressive pulmonary fibrosis
Pirfenidone	Antifibrotic and anti‐inflammatory effects involving TGF‐β‐related and other profibrotic pathways	Supported by experimental fibrosis models	A small randomized FHP trial was underpowered for its primary endpoint but reported signals for improved progression‐free survival; mixed‐ILD trials provide indirect support	Level II, low certainty	Investigational or selectively considered; evidence is insufficient to define it as universal standard therapy for FHP
IL‐17/Th17 pathway blockade	IL‐17 may promote neutrophilic inflammation, granuloma formation, and fibroblast activation	IL‐17 deletion or neutralization attenuated inflammation in several murine HP models; other experimental models suggest context‐dependent protective effects through microbial clearance	No established HP‐specific therapeutic trial demonstrating efficacy or safety	Level IV	Preclinical and investigational only
IL‐6/IL‐6R blockade	IL‐6 may contribute to inflammatory amplification, Th17 differentiation, STAT3 activation, and fibroblast signaling	Cytokine elevation and pathway activation reported mainly in experimental HP or broader fibrotic‐disease studies	No established clinical efficacy data for IL‐6 or IL‐6R blockade in HP	Level IV	Investigational only; should not be presented as an emerging clinical treatment for HP
JAK–STAT inhibition	Multiple cytokines implicated in HP signal through JAK–STAT pathways	Mechanistic rationale derived from experimental immunology, transcriptomic studies, and other immune‐mediated diseases	No established HP‐specific clinical trial demonstrating efficacy; safety and patient selection remain undefined	Level IV	Hypothesis‐generating and investigational only
Supportive care and lung‐transplant referral	Addresses functional impairment and advanced irreversible structural disease	Not applicable	Clinical practice evidence supports pulmonary rehabilitation, oxygen therapy when indicated, comorbidity management, and transplant assessment in advanced progressive disease	Practice‐based evidence	Recommended according to symptoms, physiological impairment, progression, and transplant eligibility

*Note:* Evidence levels are used descriptively rather than as a formal GRADE assessment: Level II, randomized evidence derived from small HP‐specific trials or broader progressive pulmonary fibrosis trials with an HP subgroup; Level III, nonrandomized clinical evidence from retrospective or prospective observational cohorts; Level IV, preclinical, mechanistic, or indirect evidence without established HP‐specific clinical efficacy. DLCO, diffusing capacity of the lung for carbon monoxide.

Abbreviations: FHP, fibrotic hypersensitivity pneumonitis; FVC, forced vital capacity; HP, hypersensitivity pneumonitis; PPF, progressive pulmonary fibrosis.

## 6. Conclusion and Outlook: Building a Mechanism‐Driven Paradigm for Precision Diagnosis and Treatment

This review synthesizes contemporary advances in HP, tracing the evolution from descriptive pathology toward multidisciplinary integration of exposure assessment, morphological pattern, clinical phenotype, and diagnostic confidence. The 2025 ERS/ATS statement on the classification of interstitial pneumonias distinguishes morphological patterns from final multidisciplinary diagnoses and recognizes BIP as a morphological pattern. Our description of this approach as an “etiology–morphology separation” is an interpretative synthesis, not formal terminology established specifically for HP. An unidentified inciting antigen does not, by itself, exclude HP when the combined clinical, HRCT, BAL, and pathological evidence is persuasive; equally, BIP morphology alone does not establish an HP diagnosis.

At the mechanistic level, genetic susceptibility, inhaled exposures, immune responses, epithelial injury, and maladaptive repair may interact in HP. Associations involving HLA alleles and the MUC5B promoter polymorphism support a role for host susceptibility, but do not establish that these variants consistently lower an individual threshold for disease after low‐dose antigen exposure. Human observational and single‐cell studies, together with experimental models, have identified Th1/Th17‐associated inflammation and Th2/M2‐associated profibrotic programs. We propose that changes in the relative prominence of these programs may contribute to fibrotic progression; a sequential Th1/Th17‐to‐Th2/M2 transition has not been demonstrated longitudinally in human HP. Persistent epithelial injury and fibroblast–matrix interactions may accompany increasingly treatment‐resistant remodeling, although complete fibroblast autonomy and an irreversible biological “switch” remain unproven. Compared with IPF, antigen‐associated immune injury may be more prominent among the upstream processes in FHP, while epithelial dysfunction and aberrant repair are central to the prevailing models of IPF. These are differences in emphasis within overlapping pathways rather than mutually exclusive mechanisms.

Early diagnosis remains difficult because symptoms are nonspecific, and relevant exposures may be intermittent, concealed, or difficult to identify. Potential sources include contaminated metalworking fluids and bioaerosols from musical instruments, among others. A systematic occupational, environmental, and household exposure assessment, followed by feasible source control and counseling, remains a practical priority. Novel methods for detecting cryptic exposures warrant study, but neither routine genetic screening nor unvalidated antigen‐detection assays should currently be presented as established preventive or diagnostic strategies. Similarly, patients with UIP‐like fibrosis should not be classified as having IPF solely because an inciting antigen has not been identified.

Treatment should reflect the patient’s inflammatory features, fibrotic burden, and observed disease trajectory. Antigen avoidance remains the foundational. Corticosteroids and steroid‐sparing agents such as mycophenolate mofetil or azathioprine may be considered in selected patients with clinically important inflammatory disease, although supporting HP‐specific evidence is mainly observational [109, 110]. Rituximab remains a nonstandard option supported by small observational cohorts in refractory disease; a marked response in an individual case should not be assumed to represent a predictable effect in acute exacerbations [115]. For FHP meeting criteria for progressive pulmonary fibrosis, nintedanib has support from randomized evidence that included patients with HP, whereas the evidence for pirfenidone remains preliminary [111–114]. The timing and benefit of combining immunomodulatory and antifibrotic treatment remain uncertain and should be individualized. IL‐17‐, IL‐6/IL‐6R‐, and JAK–STAT‐directed approaches are investigational in HP and have no established clinical efficacy.

Single‐cell and spatial transcriptomics are refining our understanding of cellular states and intercellular signaling, but their findings have yet to yield validated tools for routine HP care. Future research should prospectively evaluate methods for identifying difficult‐to‐detect exposures, including exhaled‐breath approaches and high‐throughput antigen assays, while establishing whether candidate biomarkers such as CCL18, CXCL13, and MMP‐7 add clinically useful information beyond exposure assessment, HRCT, lung function, and multidisciplinary diagnosis. Longitudinal human studies are especially needed to determine whether a reproducible transition from inflammation‐predominant to fibrosis‐predominant disease exists, how it relates to the treatment response, and whether progression in some patients becomes less dependent on continued antigen exposure. Resolving these questions could make stage‐adapted, mechanism‐informed management of HP more precise.

**Nomenclature**
HP: Hypersensitivity pneumonitis
ILDs: Interstitial lung diseases
ATS: American Thoracic Society
JRS: Japanese Respiratory Society
ALAT: Asociación Latinoamericana de Tórax
IPF: Idiopathic pulmonary fibrosis
CTD‐ILD: Connective tissue disease‐associated interstitial lung disease
FHP: Fibrotic hypersensitivity pneumonitis
BIP: Bronchiolocentric interstitial pneumonia
HRCT: High‐resolution computed tomography
ACCP: American College of Chest Physicians
ERS: European Respiratory Society
SNPs: Single nucleotide polymorphisms
SP: Surfactant proteins
MHC: Major histocompatibility complex
HLA: Human leukocyte antigen
AT2: Type II alveolar epithelial cells
EMT: Epithelial–mesenchymal transition
PRRs: Pattern‐recognition receptors
DAMPs: Damage‐associated molecular patterns
LPS: Lipopolysaccharide
α7‐nAChR: α7 Nicotinic acetylcholine receptor
ECM: Extracellular matrix
FMT: Fibroblast‐to‐myofibroblast transition
CTLs: Cytotoxic T cells
Treg: Regulatory T cells
TLS: Tertiary lymphoid structures
BAFF: B‐cell activating factor
APRIL: A proliferation‐inducing ligand
Bregs: Regulatory B cells
MMP: Matrix metalloproteinase
TIMP: Tissue inhibitor of metalloproteinase
SASP: Senescence‐associated secretory phenotype
UIP: Usual interstitial pneumonia
ANA: Antinuclear antibody
RF: Rheumatoid factor
NSIP: Nonspecific interstitial pneumonia
BALF: Bronchoalveolar lavage fluid.

## Author Contributions

**Chengsheng Yin**: conceptualization, visualization, writing – original draft. **Yanrui He**: writing – review and editing. **Ping Wang**: supervision, funding acquisition. **Zuojun Xu**: conceptualization, supervision, funding acquisition.

## Funding

This study was supported by the National Natural Science Foundation of China (Grant 82570097), the Beijing Municipal Natural Science Foundation (Grant 7262089), and the Integrated Research Funding for Accumulated Projects of Peking Union Medical College Hospital (Grant ZC201906243).

## Disclosure

All authors have read and agreed to the published version of the manuscript.

## Ethics Statement

This study did not involve humans or animals.

## Consent

The authors have nothing to report.

## Conflicts of Interest

The authors declare no conflicts of interest.

## Data Availability

Data sharing is not applicable to this article, as no new datasets were generated or analyzed in this study.
